# Cross-Sectional Study for Prevalence of Non-Steroidal Anti-Inflammatory Drug-Induced Gastrointestinal, Cardiac and Renal Complications in India: Interim Report

**DOI:** 10.14740/gr658w

**Published:** 2015-07-22

**Authors:** Suparna Chatterjee, Gur Prasad Dureja, Ganesh Kadhe, Amey Mane, Abhay A. Phansalkar, Sandesh Sawant, Vaibhavi Kapatkar

**Affiliations:** a Department of Pharmacology, IPGMER, Kolkata, India; b Department of Pain Medicine, New Delhi Pain Management Center, New Delhi, India; c Medical Affairs, Wockhardt Limited, Mumbai, India

**Keywords:** NSAIDs, Gastrointestinal, Renal, Cardiac, Complications, Pain management, Risk factors, Prevalence

## Abstract

**Background:**

Non-steroidal anti-inflammatory drugs (NSAIDs) are the most common therapeutic products used for the management of inflammation and pain. However, their use is associated with gastrointestinal (GI), cardiovascular and renal complications. Although prevalence data regarding NSAID-induced complications are available worldwide, but none of the study has assessed the prevalence of GI, cardiac and renal complications in India. This study aimed to assess the point prevalence of GI, cardiac and renal complications associated with the use of NSAIDs in India. The study also aimed to evaluate the association between the risk factors and GI, renal and cardiac complications in patients using NSAIDs.

**Methods:**

This prospective, cross-sectional, multi-centric study was conducted in eight medical colleges across India (North, East, West, South and Central India). Data related to GI complications including gastric, duodenal and gastroduodenal erosions/ulcers/gastritis, renal complications including acute and chronic renal failure or cardiac complications including acute coronary syndrome (ACS), acute myocardial infarction (AMI) and cardiac failure, were collected from patients.

**Results:**

The cut-off date for interim data analysis was July 7, 2014. A total of 2,140 patients out of 3,600 were enrolled from eight centers at the time of interim analysis. The NSAID-associated point prevalence of GI complications was 30.08%; cardiac complication was 42.77%; and renal complication was 27.88%.

**Conclusions:**

Results of the present interim analysis show that the prevalence of GI, cardiac and renal complications among patients is high due to exaggerated usage; however, the final analysis would provide the overall prevalence of these complications.

## Introduction

Non-steroidal anti-inflammatory drugs (NSAIDs) are mainly used for the management of inflammation and pain. Most patients with different etiologies of pain are treated with NSAIDs (up to 71.6% patients with cancer pain) as compared with other classes of drugs [1]. These agents act by inhibiting cyclooxygenase (COX) enzyme which regulates the synthesis of prostaglandins (PGs) [2]. Inhibition of COX enzyme by NSAIDs results in their analgesic, anti-inflammatory and anti-pyretic action [3]. Although NSAIDs are the most widely used therapeutic agents in the management of pain, their use is associated with gastrointestinal (GI), cardiovascular, and renal adverse events (AEs) [4]. A meta-analysis by Coxib and traditional NSAID trialists’ (CNT) collaboration reported that the use of NSAIDs increased the risk of major vascular events by 40% [5]. Another meta-analysis conducted by Scheiman reported that the risk of gastric ulcers (RR: 0.39; 95% CI: 0.31 - 0.50) and duodenal ulcers (RR: 0.20; 95% CI: 0.10 - 0.39) reduced to a significant extent, when NSAIDs were used in combination with proton pump inhibitors (PPIs) [6, 7].

The duration of NSAID use and the dose administered decide the severity of complications [3]. Lim et al in a review showed that the use of NSAIDs increased the risk of GI complications in 55-75% healthy volunteers [8]. The use of NSAIDs is inappropriate in patients with a previous history of GI events, as these agents may increase the risk of GI complications by 2.5- to 5-fold in them as compared with patients not receiving NSAIDs [9]. Traditional NSAIDs with structural components of arylacetic acids (indomethacin), arylpropionic acids (ibuprofen, ketoprofen and flurbiprofen) and anthranilates (meclofenamic acid and analogues) inhibit both isoforms of COX (COX-1 and COX-2) enzyme responsible for the synthesis of gastro-protective PGs resulting in severe GI toxicities [10]. Pareek and Chandurkar reported that the number of GI-related AEs induced by NSAIDs increased with the prolonged use of NSAIDs and a significantly higher (P = 0.053) number of GI-related AEs were observed among the patients using diclofenac as compared with aceclofenac [11]. In elderly, use of NSAIDs, including the COX-2 inhibitors, significantly increases the risk of GI bleeding. This has led American Geriatric Society (AGS) to publish a new pain management guideline stating that the use of non-selective NSAID and COX-2 inhibitors should generally not be prescribed for elderly for longer duration [12]. Co-administration of PPIs has shown a reduction in the risk of several GI complications [11, 13].

Even short-term use of NSAIDs (less than 90 days) such as ibuprofen (incidence rate ratio (IRR): 1.67; 95% CI: 1.09 - 2.57), diclofenac (IRR: 1.86; 95% CI: 1.18 - 2.92), and rofecoxib (IRR: 1.46; 95% CI: 1.03 - 2.07) was associated with increased risk of serious coronary heart disease [14]. Johnson et al demonstrated that NSAIDs increase blood pressure by up to 5 mm Hg and also antagonize the effect of antihypertensive medications such as β-blockers [15]. An increased risk of cardiac complications with concomitant use of NSAIDs has been observed among the elderly with a previous history of myocardial infarction and other cardiovascular disorders [16].

Aronoff in a review reported renal complications such as acute renal failure as a result of compromised renal blood flow and unopposed renal vasoconstriction among patients taking NSAIDs. These renal effects may occur as a result of unopposed vasoconstriction or acute interstitial nephritis due to NSAID use [17]. NSAIDs selectively inhibit renal PGs which result in renal ischemia. Elderly patients are at a higher risk of renal complications with the use of NSAIDs [18]. Kristensen et al showed that 36.1% of the patients who were on chronic renal replacement therapy had received NSAIDs within 3 years of therapy initiation [19]. Nderitu et al in a review reported that chronic renal failure progression may result from the use of high dose NSAIDs; however, a standardized guideline for calculating the optimum dose has not been defined [20].

Several studies conducted in the past have highlighted a higher risk of GI, cardiac and renal complications with the use of NSAIDs [10, 17, 21]. A higher risk of these complications has been reported among patients who are above 65 years of age, patients with a previous history of a GI and cardiac events. Also, the risk of NSAIDs-associated GI complications can be reduced with the co-administration of PPIs [9].

Although global epidemiological data regarding NSAID-induced complications are available, we planned to evaluate the prevalence of GI, cardiac and renal complications in India. This primary objective of the study was to assess the point prevalence of GI, cardiac and renal complications associated with the use of NSAIDs as a standard of care in patients across India for the first time. The other study objectives were to evaluate the association between the risk factors and GI, cardiac and renal complications in patients using NSAIDs.

## Materials and Methods

### Study characteristics

This cross-sectional, multi-centric study was conducted in eight medical colleges (Lokmanya Tilak Municipal Medical College, Mumbai; Grant Medical College, Mumbai; Government Medical College, Nagpur; Byramjee Jeejeebhoy Medical College, Pune; Nizam’s Institute of Medical Sciences, Hyderabad; Institute of Post Graduate Medical Education and Research, Kolkata; Sawai Man Singh Medical College, Jaipur and M. S. Ramaiah Institute of Technology, Bangalore) across India (North, East, West, South and Central India). Physicians from the departments of gastroenterology, cardiology, nephrology and pharmacology participated in the present study (as investigators). The study was initiated on August 16, 2013 and is presently ongoing. Written informed consent was obtained from all patients whose data were used in the study. The study protocol, informed consent form, case report form (CRF), were reviewed and approved by Institutional Ethics Committee (IEC) of respective medical colleges. The study was conducted in accordance to Good Clinical Practice (GCP) as required by the International Conference on Harmonization (ICH) guidelines, Ethical Guidelines for Biomedical Research on Human Subjects (ICMR, 2006) and Declaration of Helsinki [22].

### Patient recruitment

All data of patients were collected on a CRF by the investigators (see supplemental data for CRF). All patients in the study are being assigned a unique five-digit number by the study personnel of the pharmacology department. The first two digits of the patient identification number will represent the site code (01: site 1, 02: site 2), followed by the code for complication (GAST: patients with GI complications, CARD: patients with cardiac complications, and REN: patients with renal complications) and the remaining three digits will be a sequential number (001, 002, 003 and so on).

All patients with investigational reports of erosion/ulcer/gastritis in upper GI endoscopy; investigational reports suggestive of recent or old AMI, systolic or diastolic cardiac dysfunction/failure or ACS; or subjects who have raised serum creatinine, serum urea or low calculated creatinine clearance values suggestive of a diagnosis of renal failure (acute or chronic) are considered as “cases” while those without the above findings confirming these complications are considered as “controls”. The case/control ratio for patients with GI, cardiac and renal complications is 3:1.

### Eligibility criteria

Men or women (≥ 18 years) from outpatient department (OPD) or those admitted to the hospital for the following reasons were included in the study: admitted to gastroenterology department for upper GI endoscopy within 4 weeks prior to the study; admitted to cardiology department for one or more investigations amongst electrocardiography (ECG), tread mill test (TMT) and ECG within 3 months prior to study; and admitted to nephrology department for one or more investigations serum urea, serum creatinine and creatinine clearance within 6 months prior to study.

### Data collection

The demographic data, medical history including prior addictions to smoking, alcohol, history of NSAIDs intake including name of the medications, frequency of intake, indications of use, duration of use, whether prescribed or taken over the counter, and history of concomitant medications were collected from all patients.

Data related to GI complications including gastric, duodenal and gastroduodenal erosions/ulcers/gastritis, cardiac complications including acute coronary syndrome (ACS), acute myocardial infarction (AMI) and cardiac failure, and renal complications including acute and chronic renal failure, were collected from patients. The information on safety was also collected under the responsibility of the principal investigator.

### Sample size

The expected proportion of patients with NSAID-induced GI/cardiac/renal complications was assumed to be 0.25 (25%). An error margin of 0.025 (2.5%) and a level of confidence as 95% results in a sample size of 1,153 patients for each complication (GI, cardiac and renal). The study personnel from pharmacology department screened the medical records of gastroenterology, cardiology and nephrology departments and collecting relevant data on weekly OPD days. The subjects were enrolled for the study by designated members of the investigator’s team after visiting indoor or OPD clinics of respective departments. A total of 360 subjects were planned to be recruited from each investigational site (120 subjects each for GI, cardiac and renal complications). The total sample size will be 3,600 with case/control ratio of 3:1.

### Statistical analysis

Continuous variables (age, weight) were presented using mean and standard deviation (SD). Categorical variables (gender, presence of risk factors) were presented using frequency and percentage. Age, history of GI complications, dosage, concurrent administration of known GI toxic drugs, concurrent use of NSAIDs and history of co-morbidities were the risk factors and were considered for the analysis. A subgroup analysis was conducted based on the above specified risk factors to analyze the prevalence of NSAID-induced complications.

## Results

### Study patients

**Figure 1:**
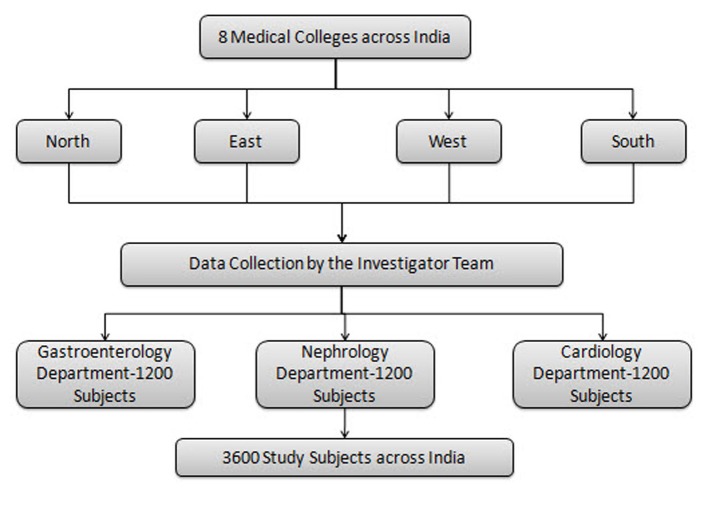
Study activity flow chart.

The proposed number of patients for the entire study is 3,600. The cut-off date for the interim analysis of the present study was July 7, 2014. Overall, 2,140 patients were enrolled up to this time and were included in this interim analysis. A total of seven investigational sites recruited 724 patients with GI complications, six investigational sites recruited 690 patients with cardiac complications, and seven investigational sites recruited 726 patients with renal complications. The study activity is presented in Figure 1.

The mean age of the patients enrolled with GI complications was 44.3 ± 14.7 years; for cardiac complications was 51.5 ± 13.4 years; and for renal complications was 45.3 ± 16.1 years. Most of the patients enrolled in the present study were male (GI: 67.6%; cardiac: 71%; and renal: 65.8%). There were 530 cases (73.8%) and 188 controls (26.9%) for GI complications; 493 cases (72.7%) and 185 controls (27.3%) for cardiac complications; and 504 cases (73.6%) and 181 controls (26.4%) for renal complications. The demographics of all the patients with all complications are presented in Table 1.

**Table 1:** Demographic Profile of Study Subjects

Complication	Gastrointestinal	Cardiac	Renal
Age (years), mean ± SD	44.3 ± 14.7	51.5 ± 13.4	45.3 ± 16.1
Gender			
Male, n (%)	483 (67.6)	486 (71)	469 (65.8)
Female, n (%)	231 (32.3)	198 (28.9)	244 (34.2)
Status			
Cases, n (%)	530 (73.8)	493 (72.7)	504 (73.6)
Controls, n (%)	188 (26.2)	185 (27.3)	181 (26.4)

### Point prevalence of NSAIDs-associated complications

**Figure 2:**
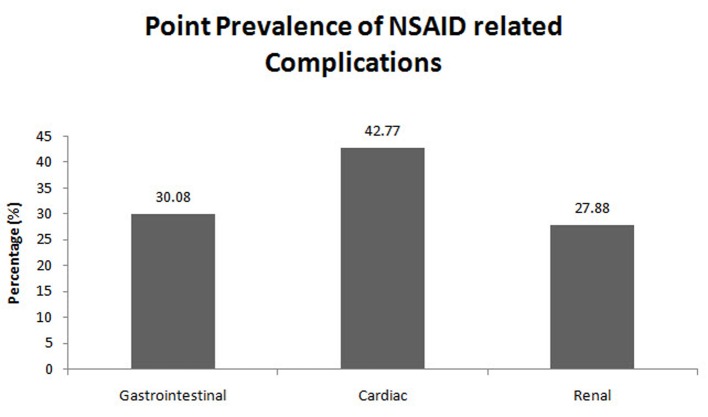
Point prevalence of NSAID-associated complications (interim analysis).

**Figure 3:**
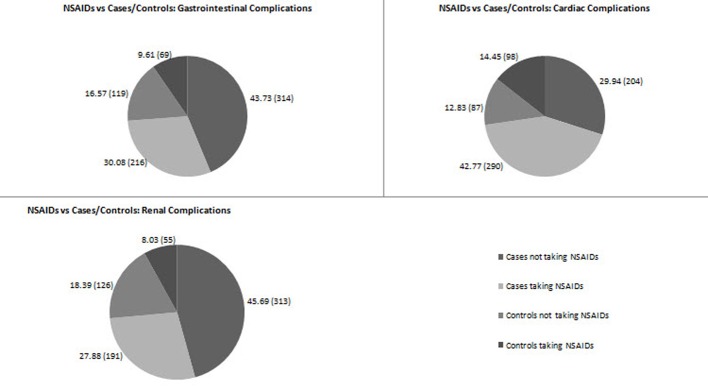
Proportion of cases and controls in GI/cardiac/renal complications (interim analysis).

Of the 724 patients enrolled in the present study with GI complications, the point prevalence of NSAID-associated GI complications was 30.08% (216 patients) (Fig. 2). The number of cases and controls among the patients taking NSAIDs was 216 (30.08%) and 69 (9.61%), respectively (Fig. 3). Of the 690 patients enrolled with cardiac complications, the point prevalence of NSAID-associated cardiac complications was 42.77% (290 patients) (Fig. 2). The number of cases among the patients taking NSAIDs was 290 (42.77%), and the number of controls was 98 (14.45%) (Fig. 3). Of the 726 patients enrolled with renal complications, the point prevalence of NSAID-associated renal complications was 27.88% (191 patients) (Fig. 2). The number of cases among the patients taking NSAIDs was 191 (27.88%) and the number of controls was 55 (8.03%) (Fig. 3).

## Discussion

The result of this interim analysis has shown the point prevalence of GI (30.08%), cardiac (42.77%) and renal complications (27.88%) with the use of NSAIDs in India. The results of this interim analysis have shown a trend of NSAIDs being used in patients with GI, cardiac and renal complications. The use of NSAIDs is exaggerated among the patient population in India. Although several other classes of analgesics are available, most patients are either prescribed NSAIDs or consume NSAIDs without the consent of their physician (OTC route).

Our previous study demonstrated that up to 55% of the patients with chronic pain received NSAIDs for pain control. Although elderly patients are at a higher risk of developing complications with the use of NSAIDs, a higher proportion of elderly (61%) have demonstrated the use of NSAIDs for pain control as per the results of our previous study [23]. Analysis of the GI, cardiac and renal complications which may result from the usage of NSAIDs is extremely important for appropriate use of NSAIDs and to standardize patient care.

Several studies conducted in the past have reported that the use of NSAIDs is associated with varying degree of complications among patients. Long-term use of NSAIDs may result in severe GI complications [24]. In addition, the risk of complications with the use of NSAIDs is higher among the elderly [25] and in patients with a history of GI [26], cardiac [14] and renal disorders [16]. The AGS advises that use of NSAIDs should be avoided in patients with abnormal renal function, a history of peptic ulcer disease or a bleeding diathesis. Further, patients should not use more than one NSAID at a time [12].

Interestingly, Simon reported that exposure to NSAIDs with a longer half life is associated with an increased risk of AEs. However; concomitant use of PPIs, H_2_ receptor antagonists and prostaglandin analogues may prove effective in reducing the risk of several GI complications [26]. However, the use of drugs such as PPIs along with NSAIDs for a longer duration is still not recommended [3]. A few studies suggest co-administration of gastro-protective agents along with NSAIDs to minimize GI-related AEs. A study conducted to evaluate the prevalence of gastroduodenal ulcers among NSAID users observed gastroduodenal mucosal injuries in 63.5% of the patients [13].

According to the current treatment guidelines with NSAIDs, use of lowest effective dose for a shorter time period is recommended. The American College of Gastroenterology, the European League Against Rheumatism, and the First International Working Party on Gastrointestinal and Cardiovascular Effects of NSAIDs and Anti-Platelet Drugs recommend the co-administration of misoprostol or gastro-protective agents such as PPls [6].

A lack of awareness regarding the prevalence rate of NSAID-induced complications among healthcare professionals and limited access to facilities are resulting in an indiscriminate use of NSAIDs for pain relief [27]. There is no specially designed study conducted across India to get relevant data about toxicities related to the use of NSAIDs. The proposed study aims to fulfill this lacuna and help educate healthcare professionals and the patients about the proper use of NSAIDs. The study would also highlight the need for appropriate pain management solutions.

In conclusion, the result of this interim analysis has shown a trend of NSAIDs being associated with most GI/cardiac/renal complications among patients. These results show that there is a need to channelize and standardize appropriate usage of NSAIDs among patients. Increasing prevalence of NSAIDs-associated GI/cardiac/renal complications may be attributed to the exaggerated use of NSAIDs among Indian population. However; the final results of this study (to be published after completion of the study in January 2015) will provide clearer evidence regarding the association of NSAIDs with GI/cardiac/renal complications. Although most studies conducted in the past have shown the efficacy of NSAIDs in relieving pain, their appropriate use is the need of hour to minimize the increasing prevalence of NSAID-related complications among patient population.
